# Long-term quality of life after hybrid robot-assisted and open Ivor Lewis esophagectomy for esophageal cancer in a single center: a comparative analysis

**DOI:** 10.1007/s00423-024-03310-2

**Published:** 2024-04-11

**Authors:** Kerstin J. Neuschütz, Lana Fourie, Nicolas Germann, Anouk Pieters, Silvio Däster, Fiorenzo V. Angehrn, Jennifer M. Klasen, Beat P. Müller-Stich, Daniel C. Steinemann, Martin Bolli

**Affiliations:** 1 Department of Visceral Surgery, Clarunis – University Digestive Health Care Center Basel, Postfach 4002 Basel, Switzerland; 2 University of Basel, Postfach 4001 Basel, Switzerland

**Keywords:** Esophageal cancer, Hybrid robot-assisted esophagectomy, Quality of life, Postoperative pain after esophagectomy

## Abstract

**Purpose:**

Due to improved survival of esophageal cancer patients, long-term quality of life (QoL) is increasingly gaining importance. The aim of this study is to compare QoL outcomes between open Ivor Lewis esophagectomy (Open-E) and a hybrid approach including laparotomy and a robot-assisted thoracic phase (hRob-E). Additionally, a standard group of healthy individuals serves as reference.

**Methods:**

With a median follow-up of 36 months after hRob-E (*n* = 28) and 40 months after Open-E (*n* = 43), patients’ QoL was assessed using the European Organization for Research and Treatment of Cancer (EORTC) QoL Questionnaire Core 30 (QLQ-C30) and the EORTC Esophagus specific QoL questionnaire 18 (QLQ-OES18).

**Results:**

Patients showed similar clinical-pathological characteristics, but hRob-E patients had significantly higher ASA scores at surgery (*p* < 0.001). Patients and healthy controls reported similar global health status and emotional and cognitive functions. However, physical functioning of Open-E patients was significantly reduced compared to healthy controls (*p* = 0.019). Operated patients reported reduced role and social functioning, fatigue, nausea and vomiting, dyspnea, and diarrhea. A trend towards a better pain score after hRob-E compared to Open-E emerged (*p* = 0.063). Regarding QLQ-OES18, hRob-E- and Open-E-treated patients similarly reported eating problems, reflux, and troubles swallowing saliva.

**Conclusions:**

The global health status is not impaired after esophagectomy. Despite higher ASA scores, QoL of hRob-E patients is similar to that of patients operated with Open-E. Moreover, patients after hRob-E appear to have a better score regarding physical functioning and a better pain profile than patients after Open-E, indicating a benefit of minimally invasive surgery.

## Introduction

Esophageal cancer is the 8th most common cancer worldwide and one of the main causes of cancer-related death [1]. Therapeutic regimens vary from local resections of lesions limited to the mucosa, to more invasive procedures such as esophagectomy, often combined with neoadjuvant radio- and chemotherapy, or chemoradiotherapy as definitive treatment, for more advanced tumor stages [1–3]. Neoadjuvant therapy followed by esophagectomy is the most frequently recommended treatment option, especially for advanced, non-metastatic esophageal cancer [4–6].

Esophagectomy is a complex and challenging surgical procedure. During the last decades, surgical approaches have evolved, and include open esophagectomy with laparotomy and thoracotomy, and fully minimally invasive procedures with laparoscopy and thoracoscopy, as well as robot-assisted esophagectomies, or combinations of these techniques [7].

Recurrence-free and overall survival of patients with esophageal cancer are generally poor [8]. However, survival rates have considerably improved throughout the past years, due to the evolution of treatment options and earlier diagnosis [9, 10]. Hence, more patients benefit from curative resections and a longer survival [11]. Therefore, long-term quality of life (QoL) is increasingly gaining importance.

The European Organization for Research and Treatment of Cancer (EORTC) Quality of Life Questionnaire Core 30 (QLQ-C30) is one of the most frequently applied tools for an objective measurement and validation of QoL in patients with cancer [12]. In addition, the EORTC Esophagus specific Quality of Life questionnaire 18 (QLQ-OES18) explicitly addresses QoL of patients after esophagectomy [11].

Systematic reviews regarding QoL of patients operated for esophageal cancer have been published in the past [13–17]. The reported results regarding postoperative quality of life vary from similar outcomes for the different surgical approaches, to beneficial results after a minimally invasive approach or a time-dependent difference [15–17]. However, they included patients treated with a large variety of techniques in a number of different institutions. Therefore, authors warned that their results should be considered cautiously, due to possible publication and selection bias [14]. Instead, data from single institutions are ideally suited for the analysis of clinical outcomes following operations performed with different surgical techniques.

During the past 20 years, our group has accumulated a profound experience in the treatment of esophageal cancer [18, 19]. Initially, open Ivor Lewis esophagectomy (Open-E) was performed [18]. Over time, a hybrid robotic-assisted Ivor Lewis esophagectomy (hRob-E) with laparotomy and thoracoscopy was adopted [19, 20]. In previous studies, we reported that similar complication rates and early oncological outcomes were observed in patients after hRob-E and Open-E surgery, although increasing numbers of patients with higher American Society of Anesthesiology (ASA) grades were treated with hRob-E [19].

Recently, QoL of patients operated with fully robotic or open Ivor-Lewis esophagectomy in a single institution was comparatively evaluated [21–24]. In this study, we are demonstrating the comparison between a hybrid minimally invasive approach, including a robot-assisted thoracic phase and an open abdominal phase, and a fully open approach. Accordingly, only the thoracic phase of the surgery was performed in a different manner.

The aim of our study is to compare the QoL of patients after hRob-E and Open-E as well as a healthy reference group, using the EORTC QLQ-C30 and EORTC QLQ-OES18 questionnaires.

## Patients and methods

### Patients

All patients with esophageal cancer who underwent potentially curative hRob-E in our clinic from October 2015 to September 2020 irrespective of neoadjuvant treatment were included in the analysis. Patients alive and without signs of tumor recurrence according to our clinic’s follow-up database were initially contacted by phone and then, following written informed consent, asked to compile the EORTC QLQ-C30 and esophagus-specific QLQ-OES18 modules. Data were analyzed in comparison with those from similarly selected patients treated with open Open-E in our institution between January 1999 and December 2010 [18] and with a standard reference group of healthy individuals [25]. The study was approved by the local ethics committee (project-ID 2021–00948).

### Surgical techniques

Open-E-treated patients were operated by three surgeons with an open abdominal and an open thoracic phase. Reconstruction was performed with a gastric conduit and an intrathoracic esophagogastric anastomosis. hRob-E-treated patients were operated with an open abdominal and a robot-assisted thoracic phase. Reconstruction was also performed with a gastric conduit and an intrathoracic esophagogastric anastomosis. All operations were performed by the same two surgeons. In summary, only the thoracic phase was conducted in a different manner, whereas the abdominal phase was conducted in the same manner in both cohorts, allowing a focused evaluation of the impact of the different approach during the thoracic phase. A comparative analysis of perioperative outcomes, including complication rates and 30-day mortality, has previously been reported [19].

### Quality of life assessment

QoL of eligible patients was evaluated using the German versions of the EORTC QLQ-C30, and of the esophagus specific QLQ-OES18 questionnaires. Both are commonly used for QoL assessment following esophagectomy for esophageal cancer [11].

The EORTC QLQ-C30 quantifies QoL of patients with malignant diseases. It includes a global health/QoL scale and five functional scales analyzing physical, role, cognitive, emotional, and social function. In addition, three symptom scales evaluate fatigue, pain, and nausea/vomiting and six single items address dyspnea, insomnia, appetite loss, constipation, diarrhea, and financial problems.

The esophagus-specific QLQ-OES18 questionnaire includes four symptom scales regarding dysphagia, eating, reflux, and pain and six single items addressing trouble swallowing saliva, choking, dry mouth, taste, coughing, and speaking.

All answers are linearly transformed in a 0–100 range. High scores in the functional scales reflect higher and thus better functional levels, whereas higher scores in a symptom or a single item scale are associated with worse QoL.

### Reference population

Data related to healthy reference populations were derived from EORTC for the EORTC QLQ-C30 questionnaire [12], and from a specific database for the QLQ-OES18 questionnaire [25].

### Study endpoints

The endpoint of this study is quality of life, represented by the categories, symptoms scales, and single items emerging from the responses to the EORTC QLQ-C30 and QLQ-OES18 questionnaires. Primarily, the data was compared for hRob-E- versus Open-E-treated patients. Secondarily, the results from patients with esophageal cancer who underwent either hRob-E or Open-E were compared to a standard population by using the same questionnaires [12, 25].

### Statistical analysis

Statistical analyses were performed using R Project for Statistical Computing (v4.1.1). Missing data consisted of 2 out of 3618 data points (< 0.001%) in the EORTC QLQ-C30 and QLQ-OES18 and was imputed with the MICE (multiple imputation by chained equation) package (v3.9.0) (https://www.rdocumentation.org.).

QoL results obtained from both EORTC questionnaires were linearly transformed into scores ranging between 0 and 100 according to the EORTC scoring manual, and healthy population values were used as a reference [12, 25]. QoL data are presented as mean values with standard deviations. We used the Shapiro–Wilk test and Levene’s assumption test to test data for normality. One-sample *t*-tests were conducted to compare the EORTC QLQ-C30 and QLQ-OES18 results of the hRob-E and Open-E study groups with the healthy reference population. To compare the results of hRob-E and Open-E groups, we conducted two-sample *t*-tests for independent samples.

## Results

### Patients’ selection and clinical-pathological characteristics

**Fig. 1:**
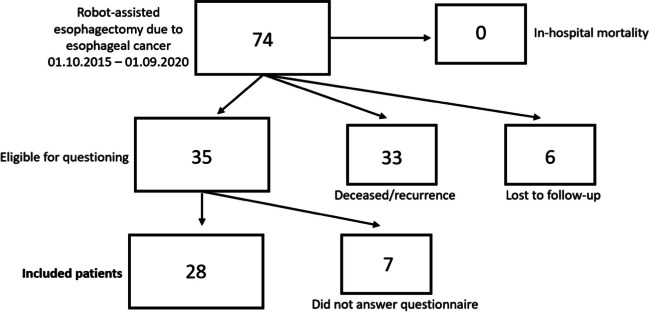
Flow diagram of patient recruitment and selection

Between October 1st 2015 and September 1st 2020, a total of 74 patients underwent hybrid robot-assisted esophagectomy (hRob-E). At the initiation of this study, 1 year after the last performed surgery, 33 patients (45%) either had a tumor recurrence or had deceased. In addition, 6 patients (8%) were lost to follow-up. Therefore, a total of 35 patients (47%) were eligible for questioning. However, 7 of them did not complete or return all questionnaires. Thus, a total of 28 patients, which fully completed and returned all questionnaires, were included in this study. The flow diagram of patient selection is shown in Fig. 1. Eligible patients after Open-E (*n* = 43) were selected according to similar criteria, as previously reported [18].

Clinical-pathological characteristics of patients which underwent hRob-E or Open-E are reported in Table 1. The median age at the time of surgery was 68 years in the robot-assisted cohort and 69 years in the open-E cohort (*p* = 0.616). In both cohorts, most patients were male—86% in hRob-E and 70% in Open-E (*p* = 0.263). In the hRob-E cohort, the majority (75%) of patients had an American Society of Anesthesiologists (ASA) score of 3, whereas in the Open-E cohort most patients (51%) had a 2 ASA score (*p* < 0.001). Regarding tumor characteristics, adenocarcinoma was more common in both groups (100% vs. 74%, *p* = 0.070) and tumors were mostly located in the distal esophagus (57% vs. 67%, *p* = 0.242). In the hRob-E cohort, most patients had a Union Internationale Contre le Cancer (UICC) stage of I or III, while patients in the Open-E cohort most frequently had a UICC stage of II (*p* = 0.741). Neoadjuvant treatment was administered to 19 patients (68%) prior to hRob-E and to 23 patients (53%) prior to Open-E (*p* = 0.313).

**Table 1:** Clinical-pathological characteristics

	hRob-E (*n* = 28)	Open-E (*n* = 43)	*p*-value
Age at surgery	68 years (61; 73)	69 years (47; 88)	0.616
Sex			
Female	4 (14%)	13 (30%)	0.263
Male	24 (86%)	30 (70%)	
ASA score			
1	0 (0%)	7 (16%)	< 0.001
2	7 (25%)	22 (51%)	
3	21 (75%)	13 (30%)	
4 or 5	0 (0%)	0 (0%)	
Unknown	1 (2%)	0 (0%)	
Tumor location			
Upper/middle esophagus	2 (7%)	5 (12%)	0.242
Distal esophagus	16 (57%)	29 (67%)	
Siewert II	10 (36%)	9 (21%)	
Histological type of cancer			
Adenocarcinoma	28 (100%)	32 (74%)	0.070
Squamous cell carcinoma	0 (0%)	11 (26%)	
Preoperative UICC stage			
I	10 (36%)	11 (26%)	0.741
II	8 (29%)	15 (35%)	
III	10 (36%)	14 (33%)	
Unknown	0 (0%)	3 (7%)	
Neoadjuvant treatment	19 (68%)	23 (53%)	0.313
Median follow-up	36 months (25; 44)	40 months (21; 135)	

*ASA* American society of Anesthesiologists, *UICC* Union Internationale Contre le Cancer

Age and median follow-up are shown as median (lower and upper quartile). Remaining data are shown as counts (percentages)

### Short-term postoperative outcomes

Among the 28 patients included in this study, the most frequent postoperative morbidity was, as commonly described in the literature [7], pneumonia (28.6%). There was one case of anastomotic insufficiency which was treated with stenting. The overall rate of morbidity was 50%. Similar complication rates were also observed among Open-E-treated patients [19].

### Assessment of general health-related quality of life

**Fig. 2:**
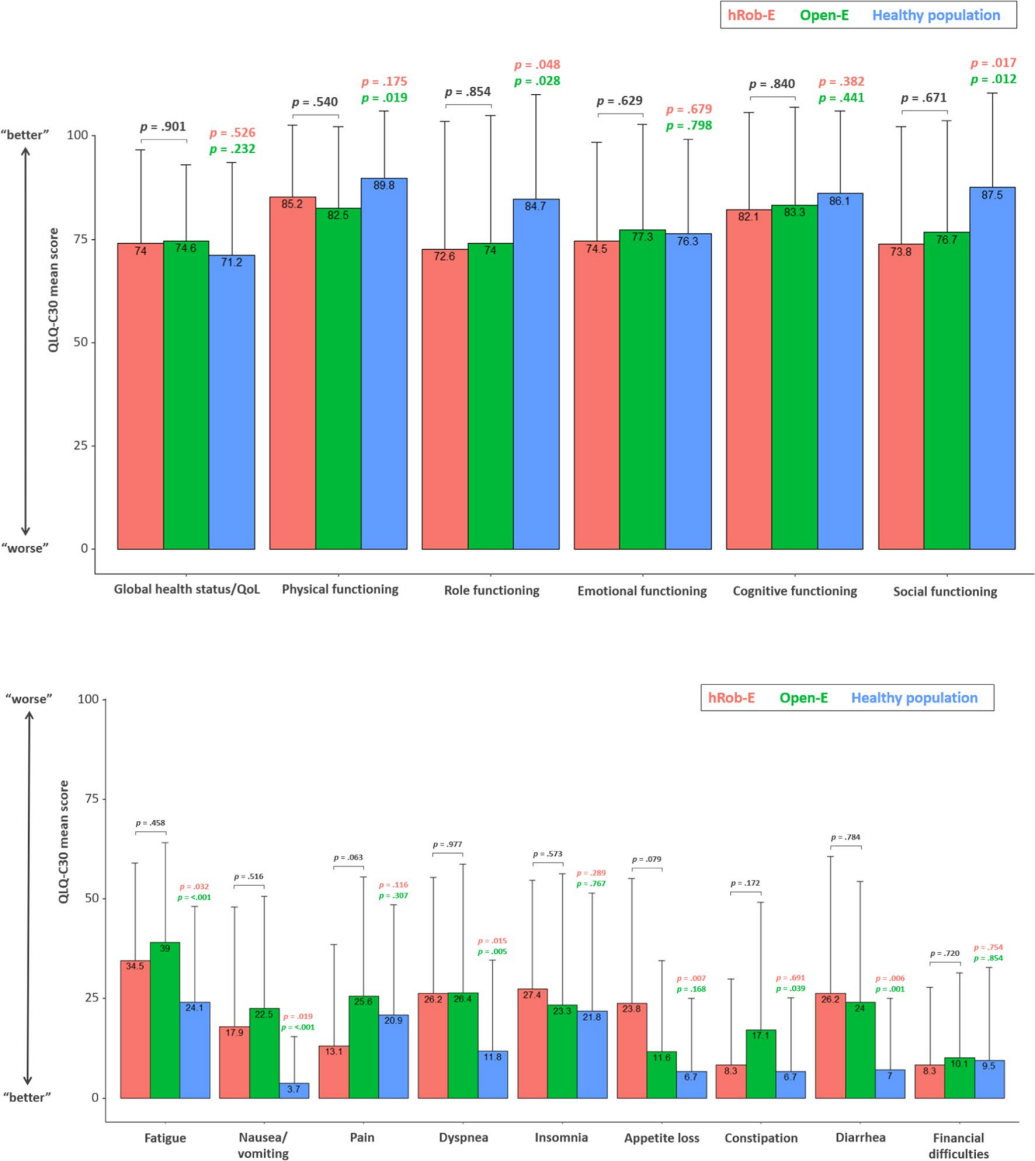
Bar graphs illustrating the results of the European Organization for Research and Treatment of Cancer (EORTC) Quality of Life Core 30 QOL-C30 questionnaire

The European Organization for Research and Treatment of Cancer Quality of Life Questionnaire Core 30 EORTC QLQ-C30 assesses the quality of life (QoL) of cancer patients, regardless of the specific existing malignancy. The mean score of the global health status was similar in the hRob-E (73.96), the Open-E (74.61), and the healthy control population (71.20) (Fig. 2, upper graph).

Considering physical functioning, patients which underwent a thoracotomy during Open-E had a significantly reduced mean score when compared to the healthy population (*p* = 0.019), whereas the mean score of hRob-E-treated patients was not significantly reduced (*p* = 0.175). However, the comparison of the mean score of both surgically treated groups revealed no significant difference (*p* = 0.540).

Regarding role functioning, the mean score was significantly reduced in both surgically treated groups with a mean of 72.62 in hRob-E and 74.03 in Open-E when compared to the general population (84.70), resulting in a *p*-value of 0.048 and 0.028, respectively. Direct comparison of hRob-E- and Open-E-treated patients’ scores revealed no significant difference. Similar results were observed regarding social functioning, with significantly reduced scores in hRob-E and Open-E patients when compared to the healthy population (*p* = 0.017 and *p* = 0.012, respectively), yet no significant difference when compared to one another (*p* = 0.671).

In contrast, regarding emotional and cognitive functioning, mean scores were similar in all three groups, revealing no significant differences.

Besides the global health status and the five functioning scales, the EORTC QLQ-C30 also includes a total of nine symptom and single item scales (Fig. 2, lower graph).

When compared to the healthy control group, the QoL questionnaire revealed worse scores for both hRob-E and Open-E regarding fatigue (*p* = 0.032 and* p* < 0.001), nausea and vomiting (*p* = 0.019 and *p* < 0.001), dyspnea (*p* = 0.015, and *p* = 0.005), and diarrhea (*p* = 0.006, and *p* = 0.001).

Patients after hRob-E experienced more appetite loss (*p* = 0.007) and Open-E-treated patients more frequently had difficulties with constipation (*p* = 0.039) when compared to the healthy population. For these items, no statistically significant difference could be shown when directly comparing hRob-E with Open-E.

Regarding insomnia, financial difficulties, and pain, the mean scores of all three groups were similar. However, a trend towards a lower pain score after hRobE, as compared to Open-E-treated patients, clearly emerged.

The assessed categories (upper panel) and symptom scales (lower panel) are marked on the *x*-axis, while mean scores are represented on the *y*-axis. Red bars refer to data from the hRob-E cohort, green bars to those from the Open-E cohort, and blue bars to those from the healthy control population.

### Specific assessment of QoL of patients with esophageal cancer

**Fig. 3:**
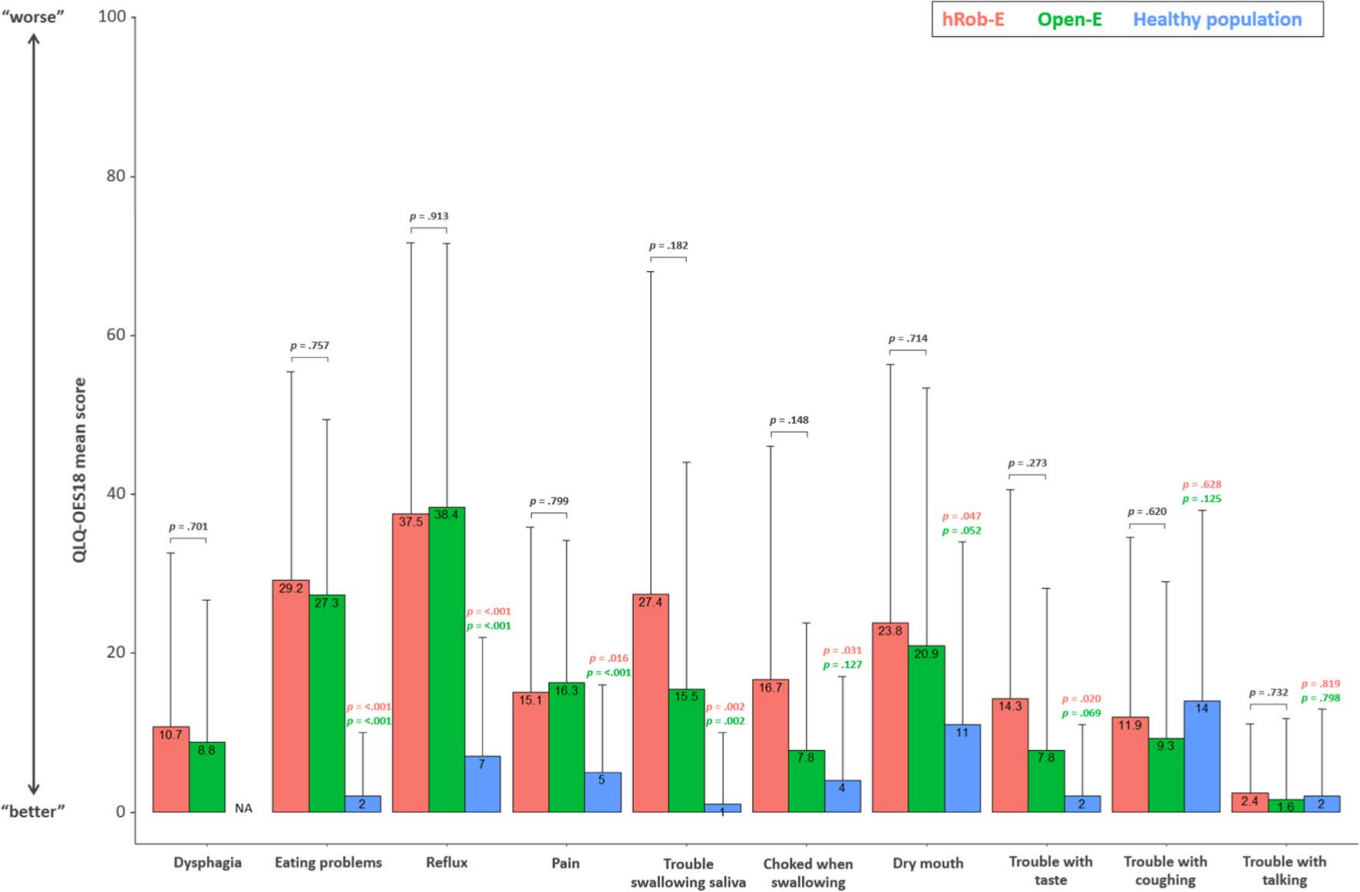
Bar graph illustrating results of the European Organization for Research and Treatment of Cancer (EORTC) Quality of Life Oesophagus specific QLQ-OES18 questionnaire

Responses to the EORTC QLQ-OES18, which was specifically designed to analyze troubles of patients with esophageal malignancies were also evaluated in detail (Fig. 3).

Patients treated for esophageal cancer with hRob-E or Open-E both had worse scores regarding eating problems (*p* < 0.001 for both groups), reflux (*p* < 0.001 for both groups), and trouble swallowing saliva (*p* = 0.002 for both groups) when compared to the healthy control population.

Regarding pain, patients of both surgically treated groups also had a worse score compared to the healthy control group (*p* = 0.016 and *p* ≤ 0.001, respectively). The mean score of patients after hRob-E was slightly, however not significantly, lower and thus better than that of patients after Open-E.

Following hRob-E, patients had more problems with choking when swallowing (*p* = 0.031), troubles with taste (*p* = 0.020), and dry mouth (0.047) compared to the healthy population. For these entities, patients after open surgery also had worse scores compared to the control population; however, the differences did not reach statistical significance.

The scores regarding trouble with coughing or talking were similar in all groups and did not differ significantly. Generally, for all items, there was no statistically significant difference when directly comparing hRob-E and Open-E (Fig. 3).

The assessed items are shown on the *x*-axis, whereas mean scores are reported on the *y*-axis. Red bars refer to data from the hRob-E cohort, green bars to those from the Open-E cohort and blue bars to those from the healthy control population.

## Discussion

Treatment of esophageal cancer is multimodal and highly challenging. During the past two decades, several important advances, including earlier diagnosis, improved surgical techniques, enhanced neoadjuvant treatment regimens, and, more recently, implementation of immunotherapy as a treatment option, have significantly contributed to ameliorate prognosis [26]. In turn, improving prognosis and survival has led to an increased focus on quality of life of esophageal cancer patients.

An early systematic review of health-related QoL following esophagectomy, performed with a variety of different techniques and comparing pre- and postoperative data, showed that pooled scores for physical function, vitality, general health perception, fatigue, dyspnea, and diarrhea 6 months after surgery were significantly worsened. Interestingly, however, emotional function was reported to be significantly improved half a year after esophagectomy [13]. More recently, another systematic review, comparing data from patients treated with minimally invasive versus open surgery, showed that while specific symptoms such as dysphagia, eating problems, and trouble swallowing saliva similarly declined after both surgical approaches, global health, and social and emotional function, as well as physical and role functions, were more frequently improved after a minimally invasive approach [14]. Especially regarding short-term QoL, minimally invasive esophagectomy has been suggested to be superior to the open approach [15, 21]. Consistently, decreased pain and esophageal symptoms, and improved emotional well-being following fully Rob-E compared to Open-E upon a 2-year follow-up, have been reported [22]. A recent multicentric study was able to show that different surgical techniques, including Ivor Lewis, McKeown, or transhiatal approaches, are associated with unique symptom profiles. Yet, overall, minimally invasive approaches led to a lower prevalence of reduced energy or activity tolerance [17]. In contrast, other studies demonstrated missing differences when comparing minimally invasive to open techniques—for both single-center and multi-center settings [16, 24].

During the past decade, we have adopted hRob-E for esophageal cancer treatment and were able to show that it is at least as safe and effective as Open-E, even when treating patients with higher ASA grades [19, 20]. Here we comparatively analyzed QoL of patients treated with hRob-E, Open-E, and healthy individuals.

Patients that received an esophagectomy for esophageal cancer in our institution showed similar results regarding the global health status when compared to healthy control subjects—irrespective of the specific surgical approach. These results are largely in agreement with those reported in recent studies including patients treated with a variety of surgical techniques [27, 28]. Furthermore, emotional and cognitive functioning scores in both surgically treated groups were similar compared to healthy individuals, suggesting that the curative intention of the esophagectomy might help patients to overcome the emotional impact of their tumor diagnosis. Explanations for these encouraging findings might include adaptive and coping mechanisms associated with recovery, and, possibly, the relation of other difficulties in comparison with the eminent matter of successful tumor treatment. Furthermore, financial difficulties do not seem to affect esophageal cancer patients after major surgery more than the healthy general population, another reassuring aspect which may be attributed to the Swiss social system. In addition, operated patients do not appear to suffer from troubles with coughing or talking any more than healthy controls, consistent, for the latter score, with the integrity of the recurrent laryngeal nerve.

Notably, however, scores reflecting role and social functioning were worse in both hRob-E- and Open-E-treated patients compared to the healthy general population. This emphasizes the fact that rehabilitation and recovery of these issues must be improved in the future. Furthermore, regarding specific symptoms, patients operated with either technique similarly reported fatigue, nausea and vomiting, dyspnea, and diarrhea. Most obviously, the role of the underlying, malignant disease as a cause of these symptoms should not be underestimated. Moreover, surgery, irrespective of its technical approach, may also play a role. For instance, nausea and vomiting may be caused by the reduced gastric reservoir or by strictures of the conduit or pyloric stenosis associated with conduit formation, while dyspnea might reflect pulmonary complications frequently associated with esophagectomy [7].

The responses to the QLQ-OES18 questionnaire revealed that eating problems, reflux, and trouble swallowing saliva were similarly experienced by patients treated with either surgical approach. These symptoms may largely be attributed to the removal of the esophagogastric junction and subsequent loss of its function as a physiological barrier. Moreover, trouble swallowing saliva may be related to dysmotility of the gastric conduit and ablation of motile esophagus. Considering that the reconstruction of intestinal continuity by forming a gastric conduit is performed similarly in both surgeries irrespective of the technical approach, the fact that the results of hRob-E- and Open-E-treated patients are similar is as anticipated.

Nonetheless, when considering that both surgically treated groups had similarly worse scores compared to healthy controls, it has to be taken into account that hRob-E patients had higher ASA scores than Open-E patients. And still, their results were not worse, which indicates that the benefit of a minimally invasive approach in hRob-E appears to balance the fact that baseline conditions of these patients were worse to begin with.

Our data is in accord with a previous study indicating that while the global health status after minimally invasive esophagectomy is not impaired, single specific symptoms such as dysphagia, reflux, eating problems, and appetite and weight loss are still reported [29]. Thus, although these symptoms appear to be controlled, since they do not influence overall QoL, their occurrence might suggest the importance and necessity of peri- and postoperative nutritional advice and guidance, accompanied by exercise training and specific health education [11, 28, 30–32].

Interestingly, regarding physical functioning, scores of patients after hRob-E were only marginally reduced compared to the healthy population, whereas those of patients after Open-E were significantly reduced. This might highlight the benefit of a hybrid minimally invasive approach, which may help to overcome the impact of major surgery on physical functioning. Since the abdominal phase was conducted in the same manner in hRob-E and Open-E, this difference may be ascribed to the avoidance of thoracotomy during hRob-E.

In this context, pain certainly represents an important parameter. In the QLQ-C30 analysis in which patients were asked whether they generally had pain, hRob-E-treated patients reported more favorable scores compared to Open-E-treated patients, although the difference failed to reach statistical significance (*p* = 0.063). This difference might be ascribed to the avoidance of a thoracotomy in hRob-E. However, in their responses to the QLQ-OES18 questionnaire, in which patients were more specifically asked whether they had pain while eating or chest pain, patients from both surgically treated cohorts similarly reported worse scores compared to the general population, consistent with the nature of the malignant disease and the necessity for surgery.

Further minor differences associated with different surgical approaches were observed. For instance, in comparison to the healthy reference population, Open-E-treated patients had significantly worse scores regarding constipation. Instead, patients treated by hRob-E had significantly worse scores regarding appetite loss. We have no obvious explanation for these differential observations. Choking when swallowing, dry mouth, and trouble with taste were also more frequently reported by hRob-E-treated patients. The underlying physio-pathological mechanisms remain unclear.

Limitations of our work should be acknowledged. Firstly, relatively low numbers of patients were included in the study. Secondly, they were treated over two decades, which might imply, in addition to the updating of surgical technology, more subtle changes in peri- and postoperative management possibly impacting QoL. On the other hand, a follow-up relatively prolonged for esophageal cancer, and the fact that these patients were treated in the same institution, thereby limiting additional variables of potential relevance, represents important strengths of our study.

## Conclusion

Taken together, our data from a single institution shows that the global health status as well as emotional and cognitive functioning are not impaired after hRob-E or Open-E for esophageal cancer.

However, the scores reflecting role and social functioning as well as specific symptoms like fatigue, nausea and vomiting, dyspnea, and diarrhea were worse in both surgically treated groups. Even though these symptoms may be attributed to the underlying malignant disease and are partially controlled, our data identifies issues that need to be addressed to improve QoL and emphasizes the importance and necessity of rehabilitation and recovery including exercise, health education, and nutritional advice.

As anticipated, scores regarding the functionality of the gastric conduit are similar after hRob-E and Open-E, which is explained by the same technique of reconstruction of intestinal continuity, irrespective of the surgical approach. However, regarding physical functioning and pain, patients after hRob-E appear to have a better profile than patients after Open-E. This might be attributed to the avoidance of thoracotomy, seen as this is the most significant technical difference between hRob-E and Open-E.

Furthermore, despite significantly higher ASA scores in hRob-E compared to Open-E patients, their outcomes were not worse. This indicates that the benefit of a minimally invasive approach may balance the fact that baseline conditions of these patients were worse to begin with.

## Data Availability

The data that support the findings of this study are available from the corresponding author upon reasonable request.
